# Admission Levels of Total Tau and β-Amyloid Isoforms 1–40 and 1–42 in Predicting the Outcome of Mild Traumatic Brain Injury

**DOI:** 10.3389/fneur.2020.00325

**Published:** 2020-05-13

**Authors:** Iftakher Hossain, Mehrbod Mohammadian, Riikka S. K. Takala, Olli Tenovuo, Leire Azurmendi Gil, Janek Frantzén, Mark van Gils, Peter J. Hutchinson, Ari J. Katila, Henna-Riikka Maanpää, David K. Menon, Virginia F. Newcombe, Jussi Tallus, Kevin Hrusovsky, David H. Wilson, Jessica Gill, Kaj Blennow, Jean-Charles Sanchez, Henrik Zetterberg, Jussi P. Posti

**Affiliations:** ^1^Division of Clinical Neurosciences, Department of Neurosurgery, Turku University Hospital, Turku, Finland; ^2^Turku Brain Injury Centre, Turku University Hospital, Turku, Finland; ^3^Department of Clinical Neurosciences, University of Turku, Turku, Finland; ^4^Department of Clinical Neurosciences, Neurosurgery Unit, University of Cambridge, Addenbrooke's Hospital, Cambridge, United Kingdom; ^5^Perioperative Services, Intensive Care Medicine and Pain Management, Turku University Hospital, Turku, Finland; ^6^Anaesthesiology, Intensive Care, Emergency Care and Pain Medicine, University of Turku, Turku, Finland; ^7^Department of Human Protein Sciences, Faculty of Medicine, University of Geneva, Geneva, Switzerland; ^8^VTT Technical Research Centre of Finland Ltd., Tampere, Finland; ^9^Division of Anaesthesia, University of Cambridge, Addenbrooke's Hospital, Cambridge, United Kingdom; ^10^Department of Radiology, Turku University Hospital, Turku, Finland; ^11^Quanterix Corporation, Lexington, MA, United States; ^12^National Institute of Nursing Research, National Institutes of Health, Bethesda, MD, United States; ^13^Department of Psychiatry and Neurochemistry, Institute of Neuroscience and Physiology, The Sahlgrenska Academy at the University of Gothenburg, Mölndal, Sweden; ^14^Clinical Neurochemistry Laboratory, Sahlgrenska University Hospital, Mölndal, Sweden; ^15^Department of Neurodegenerative Disease, UCL Institute of Neurology, Queen Square, London, United Kingdom; ^16^UK Dementia Research Institute at UCL, University College London, London, United Kingdom

**Keywords:** traumatic brain injury, total tau, β-amyloid 1-40, β-amyloid 1-42, outcome

## Abstract

**Background:** The purpose of this study was to investigate if admission levels of total tau (T-tau) and β-amyloid isoforms 1-40 (Aβ40) and 1-42 (Aβ42) could predict clinical outcome in patients with mild traumatic brain injury (mTBI).

**Methods:** A total of 105 patients with mTBI [Glasgow Coma Scale (GCS) ≥ 13] recruited in Turku University Hospital, Turku, Finland were included in this study. Blood samples were drawn within 24 h of admission for analysis of plasma T-tau, Aβ40, and Aβ42. Patients were divided into computed tomography (CT)-positive and CT-negative groups. The outcome was assessed 6–12 months after the injury using the Extended Glasgow Outcome Scale (GOSE). Outcomes were defined as complete (GOSE 8) or incomplete (GOSE < 8) recovery. The Rivermead Post Concussion Symptoms Questionnaire (RPCSQ) was also used to assess mTBI-related symptoms. Predictive values of the biomarkers were analyzed independently, in panels and together with clinical parameters.

**Results:** The admission levels of plasma T-tau, Aβ40, and Aβ42 were not significantly different between patients with complete and incomplete recovery. The levels of T-tau, Aβ40, and Aβ42 could poorly predict complete recovery, with areas under the receiver operating characteristic curve 0.56, 0.52, and 0.54, respectively. For the whole cohort, there was a significant negative correlation between the levels of T-tau and ordinal GOSE score (Spearman ρ = −0.231, *p* = 0.018). In a multivariate logistic regression model including age, GCS, duration of posttraumatic amnesia, Injury Severity Score (ISS), time from injury to sampling, and CT findings, none of the biomarkers could predict complete recovery independently or together with the other two biomarkers. Plasma levels of T-tau, Aβ40, and Aβ42 did not significantly differ between the outcome groups either within the CT-positive or CT-negative subgroups. Levels of Aβ40 and Aβ42 did not significantly correlate with outcome, but in the CT-positive subgroup, the levels of T-tau significantly correlated with ordinal GOSE score (Spearman ρ = −0.288, *p* = 0.035). The levels of T-tau, Aβ40, and Aβ42 were not correlated with the RPCSQ scores.

**Conclusions:** The early levels of T-tau are correlated with the outcome in patients with mTBI, but none of the biomarkers either alone or in any combinations could predict complete recovery in patients with mTBI.

## Introduction

Traumatic brain injury (TBI), “the silent epidemic,” will become a leading cause of disability and death globally by 2030 according to the recent estimation of the World Health Organization (1). Approximately 80–90% of all TBIs presenting to emergency departments are mild (mTBI) (2). Although most of the patients with mTBI show good recovery, a subgroup comprising 15–20% continue to have post-injury symptoms after 1 year (3). Computed tomography (CT), which is the standard tool for the assessment of acute TBI, is not sensitive enough for the long-term outcome prediction of mTBI (4, 5). Furthermore, there is still no clinically validated models for the outcome prediction following mTBI, and the performance of the tested models for mTBI are poor (6).

The process of recovery from mTBI is highly variable and individual. Importantly, there are no validated TBI biomarkers to provide objective measures of the degree of neuronal damage as well as the pathophysiological events following a TBI, which could help the clinician to evaluate the risks for incomplete recovery and to properly recognize patients who will need follow-up care (7–9). Glial fibrillary acidic protein (GFAP), ubiquitin C-terminal hydrolase-L1 (UCH-L1), and neurofilament light (NF-L) protein have been reported as promising biomarkers for the outcome prediction of mTBI (10–17).

Recently, also tau protein and β-amyloid isoforms 1-40 (Aβ40) and 1-42 (Aβ42), axon terminal biomarkers, known as the neurodegenerative biomarkers (18, 19), have been studied to explore the association between post-concussion symptoms (PCS) and neuronal damage, especially after repeated mTBIs. Tau is a microtubule-associated protein that is located in the axons of central nervous system (CNS) neurons and serves as a structural element in the axonal cytoskeleton (20–22). Total tau (T-tau) has been reported as a biomarker of injury to thin unmyelinated axons in a human post-mortem study (23). One study reported that elevated levels of plasma tau are associated with repetitive mTBIs in amateur boxers (24). Another study showed a marked increase in the plasma levels of tau in concussed professional ice hockey players (25). Serum tau levels were reported as a significant outcome predictor following severe TBI (26). In addition, admission cerebrospinal fluid (CSF) tau was correlated with long-term outcome in patients with severe TBI (27). Lately, it has been suggested that acute plasma hyperphosphorylated tau protein (P-tau) levels and the P-tau–T-tau ratio outperform T-tau levels for the outcome prediction of TBI (22).

Aβ40 (28) and Aβ42 (29, 30) reflect amyloidogenic amyloid precursor protein (APP) metabolism and have been reported as potential biomarkers of axonal damage in TBI (31). Aβ pathology, primarily consisting of aggregated Aβ42 peptides, is a histologic hallmark of Alzheimer's disease (AD) (32), and TBI has been suggested to be one of the risk factors for AD (33). Aβ pathology (amyloid plaques) have been found in boxers having dementia pugilistica (34) and in a proportion of other contact sport athletes having chronic traumatic encephalopathy (35). Although ventricular CSF levels of Aβ40 and Aβ42 were elevated during the first week after severe TBI (36), no changes in Aβ40 or Aβ42 were reported in mTBI where CSF samples were collected by lumbar puncture (37). However, for repetitive mTBI, post-injury subjective symptoms were associated with the reduction of CSF levels of Aβ40 and Aβ42 (15, 38). It has been reported that plasma levels of Aβ40 and Aβ42 do not have a value for the diagnosis and the prediction of outcome of mTBI (15, 23, 33, 35, 36). Lately, our research group has reported significant relationship between the acute plasma levels of axonal protein biomarker NF-L and the outcome in patients with mTBI (16). There are no studies correlating the admission plasma levels of the other axonal biomarkers such as Aβ40 and Aβ42 with the outcome of mTBI.

The aim of the current study was to correlate the levels of T-tau and Aβ40 and Aβ42 during the first 24 h after admission with outcome in patients with mTBI, using ultrasensitive single molecule array (Simoa) technology (39, 40). We hypothesized that these biomarkers would show some correlation with the outcome in these patients.

## Methods

### Study Population

This prospective study was a part of the EU-funded TBIcare (Evidence-based Diagnostic and Treatment Planning Solution for Traumatic Brain Injuries) project. One hundred seven (107) patients with mTBI [Glasgow Coma Scale (GCS) ≥ 13] were recruited whose blood samples were available within 24 h from the arrival to the ED of Turku University Hospital, Finland.

Inclusion criteria were lowest GCS ≥ 13, age ≥ 18 years, clinical diagnosis of TBI, and indications for acute head CT according to the NICE criteria (http://www.nice.org.uk/guidance/cg176).

Exclusion criteria were age <18 years, blast-induced or penetrating injury, chronic subdural hematoma, inability to live independently due to pre-existing brain disease, TBI or suspected TBI not needing head CT, more than 2 weeks from the injury, not living in the district thereby preventing follow-up visits, not speaking native language, or no consent received.

### Analysis of T-Tau and Aβ40 and Aβ42

Plasma T-tau was analyzed using the Human Neurology 4-Plex A assay (N4PA) on an HD-1 single molecule array (Simoa) instrument according to instructions from the manufacturer (Quanterix, Lexington, MA, USA). For T-tau, the lower limit of detection (LLoD) was 0.024 pg/ml, while the lower limit of quantification (LLoQ) was 0.053 pg/ml, and the calibration range was 0.136 pg/ml to 112 pg/ml. Plasma Aβ40 and Aβ42 concentrations were measured using a duplex Simoa immunoassay (Quanterix, Lexington, MA, USA). For Aβ40, the LLoD was 0.045 pg/ml, and the LLoQ was 0.142 pg/ml with a calibration range between 0 pg/ml to 90.0 pg/ml. For Aβ42, the LLoD was 0.142 pg/ml, and the LLoQ was 0.69 pg/ml with a calibration range between 0 and 11.0 pg/ml. The measurements were performed by board-certified laboratory technicians who were blinded to the clinical data. There were no samples below the LLoDs and LLoQs.

### TBI Severity and Outcome Grading

For the assessment of TBI severity, the lowest recorded GCS was used either at the scene of accident or emergency department (11, 17). The overall injury severity of the patients was assessed using the Injury Severity Score (ISS) (41). The duration of posttraumatic amnesia (PTA) was assessed at the outcome visit using the Rivermead method (42). The descriptive system proposed by Marshall et al. was used to analyze the CT scans (43), where class 1 corresponds with normal CT, classes 2–4 diffuse injuries, and classes 5–6 CTs with mass lesions.

### Outcome

The Extended Glasgow Outcome Scale (GOSE) was used at 6–12 months after the injury to assess the outcome (44). Outcomes were defined as complete recovery (GOSE 8) and incomplete recovery (GOSE < 8). The presence and severity of mTBI-related symptoms were assessed using the Rivermead Post Concussion Symptoms Questionnaire (RPCSQ) (45). Every patient was evaluated by the same experienced neurologist at the Turku Brain Injury Centre.

### Time Elapse

Time elapse was defined as the interval between the injury and sampling. Although the samples were obtained within 24 h of admission, they were not always drawn within 24 h after injury. Time elapse was used as a dichotomous variable, less than 24 h or more than 24 h, in the multiparameter prognostic panel analyses.

### Ethics Declarations

#### Ethics Approval and Consent to Participate

The study protocol was approved by the ethical review board of the Hospital District of South-West Finland. A written informed consent was obtained from all patients or from their next of kin.

### Statistical Analyses

Demographics of the subjects are presented as mean ± SD or percentages. The Kolmogorov–Smirnov test and visual inspection of data histograms were used to assess the normality of distribution. The levels of T-tau and Aβ40 and Aβ42 were not normally distributed, therefore, nonparametric tests were used in the statistical analyses. Data are presented as medians and interquartile range (IQR). Spearman rank correlation coefficient was used to assess the correlations between the levels of biomarkers and the outcomes. Correlations of biomarker levels with age and gender were analyzed with Pearson's and Spearman rank correlation, respectively. Spearman correlation coefficient was also used to assess the correlation between the levels of T-tau and amyloids in the whole cohort, as well as in the complete and incomplete recovery groups. Mann–Whitney *U* test was used to compare the levels of biomarkers between the outcome groups. A multivariate logistic regression analysis was performed in order to investigate if a biomarker alone or combined with other biomarkers had independent predictive power for the outcome beyond the clinical predictors. A biomarker panel analysis was used to investigate if a combination of biomarkers had better predictive ability than any biomarker alone. The regression analysis included the following variables: age, sex, educational level, ISS, worst GCS, Marshall CT classification, duration of PTA, time elapse, and the levels of T-tau and Aβ40 and Aβ42. Educational level was divided into basic school education, lower level professional, higher level professional, and academic. Marshall CT classification, sex, time elapse, and educational level were taken into account as categorical variables. Marshall class I (denoting CT-negative finding), female sex, time elapse of more than 24 h, and basic school education were used as reference categories in multivariate logistic regression. All other variables were considered to be numerical variables in the analyses. T-tau and Aβ40 and Aβ42 were used in the multivariate logistic regression models independently with the other variables and together in the same models. To study the prognostic ability of the biomarkers, area under the receiver operating characteristic (ROC) curve (AUC) was also used. AUC of 0.8 to 1.0 was considered very good; AUC of 0.7 to 0.8 was considered adequate; and AUC of 0.5 to 0.7 was considered poor (23). A value of *p* < 0.05 was considered statistically significant. For the prediction of dichotomized outcomes, cut-off values were defined using the ROC curve at the clinically compatible sensitivity >90%. For the data analyses, IBM SPSS Statistics 22 (IBM Corp, Armonk, New York, NY, USA) and MATLAB R2016b (Math Works, Natick, MA, USA) were used. Furthermore, a multiparameter prognostic panel was formed by PanelomiX toolbox (38) using clinical information (age, sex, educational levels, GCS, duration of PTA, ISS, time elapse, CT findings, and GOSE) and the admission levels of T-tau and Aβ40 and Aβ42 for the best prediction of incomplete recovery. Cut-off values were selected to ensure a sensitivity of more than 90%. For the prognostic panels, the partial AUC (pAUC) was used as a local comparative approach that focuses only on a portion of the ROC curve.

## Results

### Study Subjects

One hundred seven (107) patients with mTBI were recruited, of which GOSE score was available for 105, forming the final study population. There were 72 males (68.6%) and 33 females (31.4%), with a mean age of 47±20 years. The number of patients with CT-positive and CT-negative findings were 54 (51.4%) and 51 (48.6%), respectively. Patient characteristics are shown in Table 1. With regard to the outcome, 37 patients (35.0%) had complete recovery, 68 patients (65.0%) had incomplete recovery, and the mortality was 3.8% (*n* = 4). Among patients in whom the exact time of injury was available, the time elapse from injury to blood sampling was 28 ± 35 h (*n* = 76). In patients for whom the exact time of injury was unavailable, 11 patients were sampled within 24 h, and 18 patients were sampled after 24 h from the injury.

**Table 1:** Patient characteristic.

Age (years)	47.46 ± 20.25
Sex	
Male	72 (68.6%)
Female	33 (31.4%)
Marshall grade	
No visual pathology	51 (48.6%)
Diffuse injury	24 (22.9%)
Diffuse injury with swelling	1 (1%)
Diffuse injury with shift	1 (1%)
Mass lesions	28 (26.7%)
Pupil reactivity	
Unreactive	1 (1%)
Sluggish	2 (1.9%)
Reactive	98 (96.2%)
Missing data	4 (3.8%)
GOSE	
1	4 (3.8%)
2	0
3	6 (5.7%)
4	5 (4.8%)
5	7 (6.7%)
6	14 (13.3%)
7	32 (30.5%)
8	37 (35.0%)
Total	105 (100%)

*Demographics are reported in mean ± SD or percentages (%)*.

### The Levels of T-Tau and Outcome

**Figure 1:**
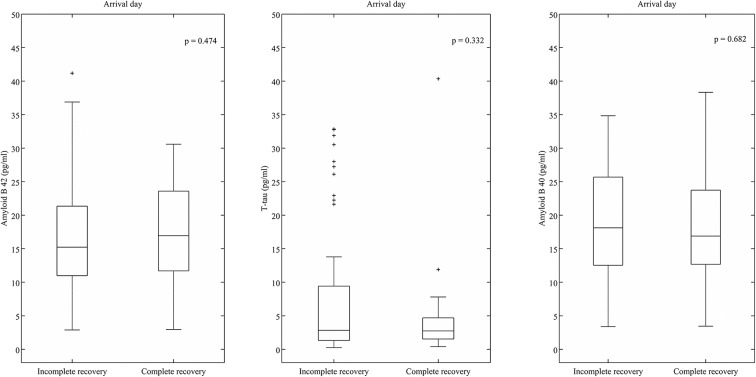
Levels of total tau (T-tau), β-amyloid isoform 1-40 (Aβ40), and β-amyloid isoform 1-42 (Aβ42) in patients with complete (GOS 8) and incomplete (GOS < 8) recovery (y axis is zoomed). Box plots represent medians in picograms per milliliter and interquartile ranges.

**Figure 2:**
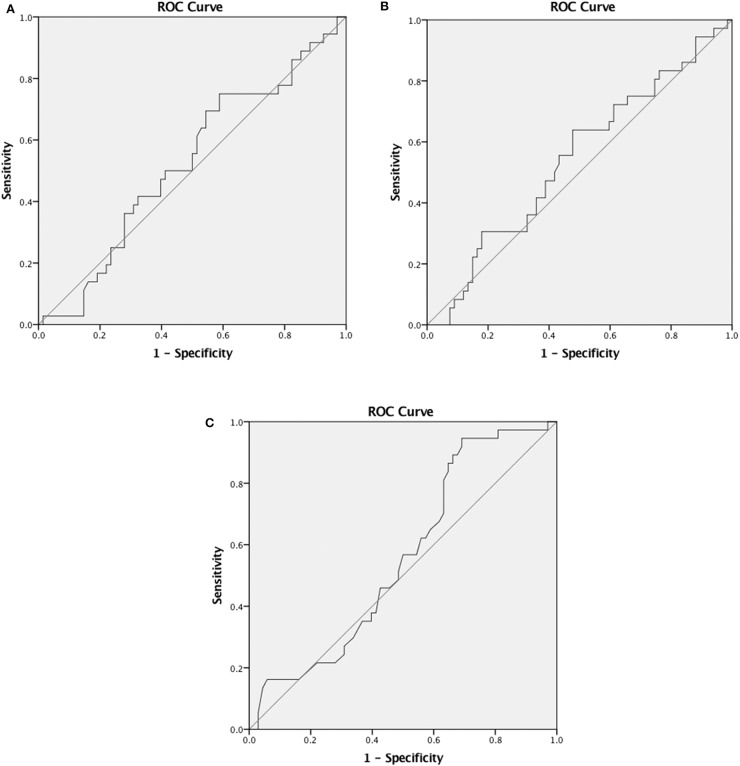
**(A)** Receiver operating characteristic (ROC) curves for predicting complete recovery (GOS 8). Area under the curve (AUC) for T-tau, 0.56 (95% CI 0.45–0.67). **(B)** ROC curves for predicting complete recovery (GOS 8). AUC for Aβ40, 0.52 (95% CI 0.41–0.64). **(C)** ROC curves for predicting complete recovery (GOS 8). AUC for Aβ42, 0.54 (95% CI 0.43–0.63).

The levels of T-tau were compared between patients with complete recovery (2.65 pg/ml, IQR 3.58 pg/ml) and incomplete recovery (2.8 pg/ml, IQR 7.5 pg/ml) (Figure 1), but significant differences were not observed. There was a significant negative correlation between the levels of T-tau and ordinal GOSE score in all patients (Spearman ρ = −0.231, *p* = 0.018) (Table 2). The level of T-tau was not able to predict the likelihood of complete recovery (AUC 0.56, 95% CI 0.45–0.67) (Figure 2A). Gender seemed to have an effect on T-tau (Table 2). The levels of T-tau did not differ between the outcome groups, and the levels of T-tau did not correlate significantly with the outcome within the CT-negative subgroup. In the CT-positive subgroup, there was a significant negative correlation between the levels of T-tau and ordinal GOSE score (Spearman ρ = −0.288, *p* = 0.035). The levels of T-tau did not correlate with the RPCSQ scores (Table 2).

**Table 2:** Correlation between biomarkers and Glasgow outcome scale extended (GOSE), gender, total PRQ, age, and RPCSQ (16 cut-off).

**Biomarker**	**GOSE**			**Gender**			**RPCSQ (total)**			**Age**			**RPCSQ (16 cut-off)**		
	**Spearman ρ**	***p*-Value**	***n***	**Spearman ρ**	***p*-Value**	***n***	**Pearson's r**	***p*-Value**	***n***	**Pearson's r**	***p*-Value**	***n***	**Pearson's r**	***p*-Value**	***n***
Amyloid β40	−0.082	0.410	104	0.034	0.731	104	−0.007	0.948	95	0.180	0.068	104	−0.007	0.946	95
Amyloid β42	0.063	0.525	103	−0.032	0.750	103	−0.015	0.889	94	0.063	0.525	103	−0.028	0.788	94
Tau	**−0.231**	**0.018**	105	**0.252**	**0.010**	105	−0.013	0.900	96	0.013	0.899	105	−0.026	0.799	96

*GOSE, Glasgow Outcome Scale extended; RPCSQ, Rivermead Post Concussion Symptoms Questionnaire. Statistically significant findings are in bold*.

#### The Levels of Aβ40 and Aβ42 and Outcome

The levels of Aβ40 were not significantly different between patients with complete (16.9 pg/ml, IQR 12.76 pg/ml) and incomplete recovery (17.42 pg/ml, IQR 12.65 pg/ml). The levels of Aβ42 were also not significantly different between patients with complete (16.94 pg/ml, IQR 12.36 pg/ml) and incomplete recovery (15.23 pg/ml, IQR 10.61 pg/ml) (Figure 1). There was no significant correlation between the levels of Aβ40 and Aβ42 and the GOSE score (Table 2). Aβ40 and Aβ42 were not able to predict the likelihood of complete recovery (AUC 0.52, 95% CI 0.41–0.64 and AUC 0.54, 95% CI 0.43–0.63, respectively) (Figures 2B,C).

When patients were divided into CT-positive and CT-negative subgroups, the levels of Aβ40 and Aβ42 did not differ between the outcome groups, nor did the levels correlate significantly with the outcome within these subgroups. The levels of Aβ40 and Aβ42 did not correlate with the RPCSQ scores (Table 2).

### Combining T-Tau, Aβ40, and Aβ42

Using conventional multivariate logistic regression model, Aβ40 and Aβ42 were not able to predict outcome independently or together with T-tau, or vice versa. We also used the Panelomix tool for evaluating the capacity of these three biomarkers in predicting incomplete recovery. When setting the sensitivity to >90%, we found that the optimal sensitivity and specificity was 92.5% (95% CI, 85.1–98.5) and 27.8% (95% CI, 13.9–41.7), respectively (Supplementary Figure 1), when the levels of at least two out of T-tau, Aβ40, and Aβ42 were above 0.55, 20.26, and 23.9 pg/ml, respectively.

### Correlation Among the Levels of T-tau, Aβ40, and Aβ42

For the whole population, as well as complete and incomplete recovery subgroups, the levels of T-tau and Aβ40 and Aβ42 were not significantly correlated with each other.

### Best Multiparameter Panel for Outcome Prediction

We also tried to find the best combination for predicting the outcome by combining clinical variables, biomarker levels, and taking into consideration the time from injury to sampling. The best available panel found was for the levels of T-tau taken more than 24 h from the injury and combined with age and ISS. This panel had a sensitivity of 90.8% (95% CI, 83.1–96.9) and a specificity of 57.1% (95% CI, 40–74.3), provided that at least two of these three variables were above their cut-off values (22.5 years for age, 3.5 for ISS, and 12.84 pg/ml for T-tau) (Supplementary Figure 2).

## Discussion

This prospective, observational study including patients with CT-positive and CT-negative mTBI investigated the performance of the blood protein biomarkers T-tau, Aβ40, and Aβ42 for the outcome prediction during the first 24 h after admission, utilizing modern highly sensitive immunoassays in a well-characterized cohort. We found that T-tau was significantly correlated with the outcome in the whole population as well as in the subgroup of patients with CT-positive mTBI. However, the levels of T-tau, Aβ40, and Aβ42 were not significantly different between the patients with complete and incomplete recovery, and the levels of T-tau, Aβ40, and Aβ42 were not able to give any useful prediction about the likelihood of complete recovery. Moreover, none of the biomarkers was correlated with the symptom severity as assessed with the RPCSQ scores. Yet, a multiparameter panel method suggested that levels of T-tau may have predictive value when sampled >24 h from the injury and combined with age and ISS, obtaining a sensitivity of 90.8% and a specificity of 57.1% for predicting incomplete recovery.

Earlier studies reported that serum tau had limited value for the diagnosis of intracranial injury and the outcome prediction of mTBI (46, 47), which is in agreement with our results. Recently, TRACK-TBI investigators used another high-sensitive assay platform and reported that acute P-tau levels and the P-tau–T-tau ratio outperformed T-tau levels in the outcome prediction of TBI (22). As only the levels of T-tau were measured in our study, the results might have been different if also P-tau was measured. Since tau is mainly expressed in unmyelinated cortical axons (15), the inability of the admission levels of plasma T-tau to differentiate complete and incomplete recovery may support the concept that in most of the cases of mTBI, mainly subcortical myelinated axons of the white matter are injured (15, 16, 48). Another possible explanation is that the eventual injury of cortical axons is a slower process, not reflected in blood levels of T-tau during the time frame used in this study.

Our study findings of Aβ40 and Aβ42 are in line with the results of the previous studies (15, 23, 33, 35, 36), where the levels of Aβ40 and Aβ42 did not correlate with the outcome as well as the levels were unable to predict complete and incomplete recovery.

A recent study reported that there was no significant relationship between the plasma levels of T-tau and Aβ42 and neurocognitive tests following mTBI (49). The study used late levels of T-tau, which is why our results cannot be compared with those data.

There are limitations in our study. First, we had data on T-tau and Aβ40 and Aβ42 available only at a single timepoint—within 24 h after admission. A kinetic study with serial sampling would allow estimation of the total efflux of a biomarker and timing of the peak values, which could reveal more information about the outcome prediction abilities of the studied biomarkers (50). Tau has been reported to be a long-term biomarker having the peak value within the first hour after the initial injury and a second peak after 36 h following mTBI (25). Aβ42 becomes significantly elevated within the first 24 h after injury and remains quite stable for ca. 6 days (18), although, there are contraindicatory studies reporting no significant elevation of Aβ40 and Aβ42 following mTBI (48). Indeed, we found that the levels of T-tau seemed to perform best when taken >24 h from the injury and combined with clinical variables. The outcome prediction abilities of the studied blood biomarkers could be negatively driven by the variability in timing of sample collection in relation to injury between patients. The most accurate diagnostic time windows for the biomarkers might have been missed; however, the time from injury to sampling was taken into account as a covariate in the analysis. Second, the variability in assessing the GOSE between 6 and 12 months after the injury should be considered as a limitation of the study. This limitation has been elaborately discussed in one of our recently published biomarker studies utilizing the same study cohort (16). Third, our patients with mTBI had more severe injuries than an average mTBI population who are seen at the ED. This is because there was a recruitment bias favoring those patients who required in-hospital treatment. This is why many patients of our mTBI cohort had abnormalities on CT. In addition, some patients—although having GCS in the mild category—had PTA for >24 h, which according to many classifications indicate a more severe TBI. These issues reflect the problems in defining an acute TBI by severity, nicely shown also in the CENTER-TBI study (51), where about one-third of cases treated at the ICU had mTBI based on GCS (52). Thus, when interpreting our results, the nature of our study population has to be taken into account. Additionally, in our study, the duration of PTA was assessed retrospectively at the outcome visit, which is considered to be less reliable than prospective evaluation. When comparing our results with earlier studies, it is important to note that none of our patients had a sports-related repetitive injury as the injury mechanism, and CSF samples were not collected.

A strength of our study is the use of ultrasensitive single molecule array (Simoa) technology. Especially for T-tau, the concentrations are very low in the peripheral blood and are thus almost impossible to measure precisely by most of the immunoassays (18). In addition, our patient cohort was prospectively collected and well characterized.

In this study, we studied biomarkers that mainly originate from axon terminals. However, they apparently represent a different kind of axonal damage, and thus, we sought to investigate their outcome prediction ability in a panel analysis. Since mTBI is a complex cascade of neurometabolic changes (25), therefore, developing a prediction model including the blood biomarkers of different cellular origins is an emerging need. It has recently been reported that panels of biomarkers from different cellular origins outperform single proteins' ability to detect patients with a need for head CT scanning after TBI (53). It has also been reported that a serum biomarker panel consisting of proteins of different cellular origins improved outcome prediction in TBI, where 70% of the cohort had severe TBI (50).

## Conclusions

The main finding of the current study was that the admission levels of T-tau were significantly correlated with the outcome in patients with mTBI. Neither T-tau, Aβ40, or Aβ42 alone or their different combinations could predict complete recovery in patients with mTBI. Our study showed that T-tau may have potential in outcome prediction of mTBI, but more studies are needed using larger sample sizes, serial sampling method, and possibly including P-tau and P-tau/T-tau ratio. Panels of biomarkers of different cellular origins are recommended to be utilized as they appear to outperform single biomarkers in outcome prediction.

## Data Availability Statement

The datasets generated for this study are available on request to the corresponding author.

## Ethics Statement

The studies involving human participants were reviewed and approved by Ethics Committee of Southwest Finland. The patients/participants provided their written informed consent to participate in this study.

## Author Contributions

IH, JP, RT, MM, and OT conceived and designed the study. JP, RT, AK, H-RM, JT, and OT recruited the patients. JP, RT, AK, H-RM, JT, IH, and OT designed the data collection at Turku University Hospital. MM conducted the statistical analyses with contributions from IH, LA, and J-CS. JG, HZ, and KB supervised the biomarker analyses. IH drafted the manuscript with critical contributions from RT, OT, and JP. MM, LA, JF, MG, PH, AK, H-RM, DM, VN, JT, KH, DW, JG, KB, J-CS, and HZ contributed to the revision of the manuscript. IH and JP take the responsibility for the paper as whole.

## Conflict of Interest

RT has received speakers fee from Abbott, Fresenius-Kabi, Orion and UCB, conference funding from Pfizer and Steripolar and is stockholder of Orion. DM reports collaborative research or consultancy agreements with GlaxoSmithKline Ltd; Ornim Medical; Shire Medical; Calico Inc; Pfizer Ltd; Pressura Ltd; Glide Pharma Ltd; NeuroTraumaSciences LLC; Lantasman AB. HZ has served at advisory boards for Roche Diagnostics, Wave, Samumed and CogRx, has participated in symposia sponsored by Alzecure and Biogen, and is a co-founder of Brain Biomarker Solutions in Gothenburg AB, a GU Ventures-based platform company at the University of Gothenburg. KB has served as a consultant or at advisory boards for Alzheon, BioArctic, Biogen, Eli Lilly, Fujirebio Europe, IBL International, Merck, Novartis, Pfizer, and Roche Diagnostics, and is a co-founder of Brain Biomarker Solutions in Gothenburg AB, a GU Ventures-based platform company at the University of Gothenburg. JP has received speaker's fees from Orion corporation and Finnish Medical Association and a travel grant from Stryker Corporation. The remaining authors declare that the research was conducted in the absence of any commercial or financial relationships that could be construed as a potential conflict of interest. The reviewer JY declared a past co-authorship with several of the authors KH, DW to the handling editor.

## References

[1] Mathers CD Loncar D. Projections of global mortality and burden of disease from 2002 to 2030. PLoS Med. (2006) 3:e442. 10.1371/journal.pmed.0030442 17132052 PMC1664601

[2] Levin HS Diaz-Arrastia R. R. Diagnosis, prognosis, and clinical management of mild traumatic brain injury. Lancet Neurol. (2015) 14:506–17. 10.1016/S1474-4422(15)00002-2 25801547

[3] Carroll LJ Cassidy JD Cancelliere C Côté P Hincapié CA Kristman VL. Systematic review of the prognosis after mild traumatic brain injury in adults: cognitive, psychiatric, and mortality outcomes: results of the international collaboration on mild traumatic brain injury prognosis. Arch Phys Med Rehabil. (2014) 95:S152–S73. 10.1016/j.apmr.2013.08.300 24581903

[4] Jagoda AS Bazarian JJ Bruns JJ Cantrill SV Gean AD Howard PK. Clinical policy: neuroimaging and decisionmaking in adult mild traumatic brain injury in the acute setting. Ann Emerg Med. (2008) 52:714–48. 10.1016/j.annemergmed.2008.08.021 19027497

[5] Mittl RL Grossman RI Hiehle JF Hurst RW Kauder DR Gennarelli TAW. Prevalence of mR evidence of diffuse axonal injury in patients with mild head injury and normal head cT findings. Am J Neuroradiol. (1994) 15:1583–9. 7985582 PMC8334423

[6] Lingsma HF Yue JK Maas AIR Steyerberg EW Manley GT Cooper SR. Outcome prediction after mild and complicated mild traumatic brain injury: external validation of existing models and identification of new predictors using the tRACK-TBI pilot study. J Neurotrauma. (2015) 32:83–94. 10.1089/neu.2014.3384 25025611 PMC4291219

[7] Dadas A Washington J Diaz-Arrastia R Janigro D. Biomarkers in traumatic brain injury (TBI): a review. Neuropsychiatr Dis Treat. (2018) 14:2989–3000. 10.2147/NDT.S125620 30510421 PMC6231511

[8] Bogoslovsky T Gill J Jeromin A Davis C Diaz-Arrastia R. Fluid biomarkers of traumatic brain injury and intended context of use. Diagnostics. (2016) 6:37. 10.3390/diagnostics6040037 27763536 PMC5192512

[9] Wang KK Yang Z Zhu T Shi Y Rubenstein R Tyndall JAT. An update on diagnostic and prognostic biomarkers for traumatic brain injury. Expert Rev. Mol. Diagn. (2018) 18:165–80. 10.1080/14737159.2018.1428089 29338452 PMC6359936

[10] Diaz-Arrastia R Wang KKW Papa L Sorani MD Yue JK Puccio AMJ. Acute biomarkers of traumatic brain injury: relationship between plasma levels of ubiquitin c-terminal hydrolase-L1 and glial fibrillary acidic protein. J. Neurotrauma. (2014) 31:19–25. 10.1089/neu.2013.3040 23865516 PMC3880090

[11] Takala RSK Posti JP Runtti H Newcombe VF Outtrim J Katila AJ. Glial fibrillary acidic protein and ubiquitin c-Terminal hydrolase-L1 as outcome predictors in traumatic brain injury. World Neurosurg. (2016) 87:8–20. 10.1016/j.wneu.2015.10.066 26547005

[12] Papa L Brophy GM Welch RD Lewis LM Braga CF Tan CN. Time course and diagnostic accuracy of glial and neuronal blood biomarkers gFAP and uCH-L1 in a large cohort of trauma patients with and without mild traumatic brain injury. JAMA Neurol. (2016) 73:551. 10.1001/jamaneurol.2016.0039 27018834 PMC8805143

[13] Welch RD Ayaz SI Lewis LM Unden J Chen JY Mika VH Ability of serum glial fibrillary acidic protein, ubiquitin c-Terminal hydrolase-L1, and s100B to differentiate normal and abnormal head computed tomography findings in patients with suspected mild or moderate traumatic brain injury. J Neurotrauma. (2016) 33:203–14. 10.1089/neu.2015.4149 26467555 PMC4722555

[14] Shahim P Zetterberg H Tegner Y Blennow K. Serum neurofilament light as a biomarker for mild traumatic brain injury in contact sports. Neurology. (2017) 88:1788–94. 10.1212/WNL.0000000000003912 28404801 PMC5419986

[15] Shahim P Tegner Y Gustafsson B Gren M Ärlig J Olsson M Lehto N. Neurochemical aftermath of repetitive mild traumatic brain injury. JAMA Neurol. (2016) 73:1308–15. 10.1001/jamaneurol.2016.2038 27654934

[16] Hossain I Mohammadian M Takala RSK Tenovuo O Lagerstedt L Ala-Seppälä H- Early levels of glial fibrillary acidic protein and neurofilament light protein in predicting the outcome of mild traumatic brain injury. J Neurotrauma. (2019) 2018:5952 10.1089/neu.2018.5952 30489229

[17] Gan ZS Stein SC Swanson R Guan S Garcia L Mehta D. Blood biomarkers for traumatic brain injury: a Quantitative assessment of diagnostic and prognostic accuracy. Front Neurol. (2019) 10:446. 10.3389/fneur.2019.00446 31105646 PMC6498532

[18] Mondello S Buki A Barzo P Randall J Provuncher G Hanlon D. CSF and plasma amyloid-β temporal profiles and relationships with neurological status and mortality after severe traumatic brain injury. Sci Rep. (2014) 4:6446. 10.1038/srep06446 25300247 PMC4192636

[19] Jack CR Wiste HJ Therneau TM Weigand SD Knopman DS Mielke MMC. Associations of amyloid, tau, and neurodegeneration biomarker profiles with rates of memory decline among individuals without dementia. JAMA. (2019) 321:2316 10.1001/jama.2019.7437 31211344 PMC6582267

[20] Binder LI Frankfurter A Rebhun LI. The distribution of tau in the mammalian central nervous system. J Cell Biol. (1985) 101:1371–8. 10.1083/jcb.101.4.1371 3930508 PMC2113928

[21] Olivera A Lejbman N Jeromin A French LM Kim H. S.. Peripheral total tau in military personnel who sustain traumatic brain injuries during deployment. JAMA Neurol. (2015) 72:1109. 10.1001/jamaneurol.2015.1383 26237304

[22] Rubenstein R Chang B Yue JK Chiu A Winkler EA Puccio AM. Comparing plasma phospho tau, total tau, and phospho tau-Total tau ratio as acute and chronic traumatic brain injury biomarkers. JAMA Neurol. (2017) 74:1063. 10.1001/jamaneurol.2017.0655 28738126 PMC5710183

[23] Zetterberg H Smith DH Blennow K. Biomarkers of mild traumatic brain injury in cerebrospinal fluid and blood. Nat Rev Neurol. (2013) 9:201–10. 10.1038/nrneurol.2013.9 23399646 PMC4513656

[24] Neselius S Zetterberg H Blennow K Randall J Wilson D Marcusson J. Olympic boxing is associated with elevated levels of the neuronal protein tau in plasma. Brain Inj. (2013) 27:425–33. 10.3109/02699052.2012.750752 23473386

[25] Shahim P Tegner Y Wilson DH Randall J Skillbäck T Pazooki D. Blood biomarkers for brain injury in concussed professional ice hockey players. JAMA Neurol. (2014) 71:684. 10.1001/jamaneurol.2014.367 24627036

[26] Liliang PC Liang CL Weng HC Chen HJ Chuang JH. Tau proteins in serum predict outcome after severe traumatic brain injury. J Surg Res. (2010) 160:302–7. 10.1016/j.jss.2008.12.022 19345376

[27] Ost M Nylén K Csajbok L Ohrfelt AO Tullberg M Wikkelsö C. Initial cSF total tau correlates with 1-year outcome in patients with traumatic brain injury. Neurology. (2006) 67:1600–4. 10.1212/01.wnl.0000242732.06714.0f 17101890

[28] Bazarian JJ Blyth BJ He H Mookerjee S Jones C Kiechle K. Classification accuracy of serum apo a-I and s100B for the diagnosis of mild traumatic brain injury and prediction of abnormal initial head computed tomography scan. J Neurotrauma. (2013) 30:1747–54. 10.1089/neu.2013.2853 23758329 PMC4047844

[29] Bogoslovsky T Wilson D Chen Y Hanlon D Gill J Jeromin A. Increases of plasma levels of glial fibrillary acidic protein, tau, and amyloid beta up to 90 days after traumatic brain injury. J Neurotrauma. (2017) 34:66–73. 10.1089/neu.2015.4333 27312416 PMC5198034

[30] Blennow K Nellgård B. Amyloid beta 1-42 and tau in cerebrospinal fluid after severe traumatic brain injury. Neurology. (2004) 62:159; author reply 159–60. 10.1212/WNL.62.1.159 14718730

[31] Johnson VE Stewart W Smith DH. Axonal pathology in traumatic brain injury. Exp Neurol. (2013) 246:35–43. 10.1016/j.expneurol.2012.01.013 22285252 PMC3979341

[32] Murphy MP LeVine H III. Alzheimer's disease and the amyloid-beta peptide. J Alzheimers. Dis. (2010) 19:311–23. 20061647 10.3233/JAD-2010-1221 PMC2813509

[33] Tsitsopoulos PP Marklund N. Amyloid-β peptides and tau protein as biomarkers in cerebrospinal and interstitial fluid following traumatic brain injury: a Review of experimental and clinical studies. Front Neurol. (2013) 4:79. 10.3389/fneur.2013.00079 23805125 PMC3693096

[34] Roberts GW Allsop D Bruton C. The occult aftermath of boxing. J Neurol. (1990) 53:373–8. 10.1136/jnnp.53.5.373 2191084 PMC488051

[35] McKee AC Cairns NJ Dickson DW Folkerth RD Keene CD Litvan I. The first NINDS/NIBIB consensus meeting to define neuropathological criteria for the diagnosis of chronic traumatic encephalopathy. Acta Neuropathol. (2016) 131:75–86. 10.1007/s00401-015-1515-z 26667418 PMC4698281

[36] Olsson A Csajbok L Ost M Höglund K Nylen K Rosengren L. Marked increase of beta-amyloid(1-42) and amyloid precursor protein in ventricular cerebrospinal fluid after severe traumatic brain injury. J Neurol. (2004) 251:870–6. 10.1007/s00415-004-0451-y 15258792

[37] Neselius S Brisby H Theodorsson A Blennow K Zetterberg H Marcusson. CSF-biomarkers in olympic boxing: diagnosis and effects of repetitive head trauma. PLoS ONE. (2012) 7:e33606. 10.1371/journal.pone.0033606 22496755 PMC3319096

[38] Shahim P Tegner Y Marklund N Höglund K Portelius E Brody DL. Astroglial activation and altered amyloid metabolism in human repetitive concussion. Neurology. (2017) 88:1400–7. 10.1212/WNL.0000000000003816 28283595 PMC5386435

[39] Wilson DH Rissin DM Kan CW Fournier DR Piech T Campbell TG. The simoa hD-1 analyzer. J Lab Autom. (2016) 21:533–47. 10.1177/2211068215589580 26077162

[40] Kuhle J Barro C Andreasson U Derfuss T Lindberg R Sandelius Å. Comparison of three analytical platforms for quantification of the neurofilament light chain in blood samples: ELISA, electrochemiluminescence immunoassay and simoa. Clin Chem Lab Med. (2016) 54:1655–61. 10.1515/cclm-2015-1195 27071153

[41] Baker SP O'Neill B Haddon W Long WB. The injury severity score: a method for describing patients with multiple injuries and evaluating emergency care. J Trauma. (1974) 14:187–96. 10.1097/00005373-197403000-00001 4814394

[42] King NS Crawford S Wenden FJ Moss NE Wade DT Caldwell. Measurement of post-traumatic amnesia: how reliable is it? J Neurol Neurosurg Psychiatry. (1997) 62:38–42. 10.1136/jnnp.62.1.38 9010398 PMC486693

[43] Marshall LF Marshall SB Klauber MR Van Berkum Clark M Eisenberg H Jane JA. The diagnosis of head injury requires a classification based on computed axial tomography. J Neurotrauma. (1992) 9(Suppl 1):S287–92. 1588618

[44] Wilson JTL Pettigrew LEL Teasdale GM. Structured interviews for the glasgow outcome scale and the extended glasgow outcome scale: guidelines for their use. J Neurotrauma. (1998) 15:573–85. 10.1089/neu.1998.15.573 9726257

[45] King NS Crawford S Wenden FJ Moss NE Wade DT. The rivermead post concussion symptoms questionnaire: a measure of symptoms commonly experienced after head injury and its reliability. J Neurol. (1995) 242:587–92. 10.1007/BF00868811 8551320

[46] Kavalci C Pekdemir M Durukan P Ilhan N Yildiz M Serhatlioglu S. The value of serum tau protein for the diagnosis of intracranial injury in minor head trauma. Am J Emerg Med. (2007) 25:391–5. 10.1016/j.ajem.2006.10.008 17499655

[47] Bulut M Koksal O Dogan S Bolca N Ozguc H Korfali E. Tau protein as a serum marker of brain damage in mild traumatic brain injury: preliminary results. Adv Ther. (2006) 23:12–22. 10.1007/BF02850342 16644603

[48] Zetterberg H Smith DH Blennow K. Neurochemical aftermath of amateur boxing. JAMA Neurol. (2006) 63:1277–80. 10.1001/archneur.63.9.1277 16966505

[49] Lippa SM Yeh P. H. Gill J French LM Brickell TA Lange RT. Plasma tau and amyloid are not reliably related to injury characteristics, neuropsychological performance, or white matter integrity in service members with a history of traumatic brain injury. J Neurotrauma. (2019) 36:2190–9. 10.1089/neu.2018.6269 30834814 PMC6909749

[50] Thelin E Al Nimer F Frostell A Zetterberg H Blennow K Nyström H. A serum protein biomarker panel improves outcome prediction in human traumatic brain injury. J Neurotrauma. (2019) 36:2850–62. 10.1089/neu.2019.6375 31072225 PMC6761606

[51] Maas AIR Menon DK Adelson PD Andelic N Bell MJ Belli A. Traumatic brain injury: integrated approaches to improve prevention, clinical care, and research. Lancet Neurol. (2017) 16:987–1048. 10.1016/S1474-4422(17)30371-X 29122524

[52] Steyerberg EW Wiegers E Sewalt C Buki A Citerio G De Keyser V. Case-mix, care pathways, and outcomes in patients with traumatic brain injury in CENTER-TBI: a European prospective, multicentre, longitudinal, cohort study. Lancet Neurol. (2019) 18:923–34. 10.1016/S1474-4422(19)30232-7 31526754

[53] Posti JP Takala RSK Lagesrstd L Dickens AM Hossain I Mohammadian M. Correlation of blood biomarkers and biomarkers panels with traumatic findings on computed tomography after traumatic brain injury. J Neurotrauma. (2019) 36:2178–89. 10.1089/neu.2018.6254 30760178 PMC6909751

[54] Hossain I Mohammadian M Takala RSK Tenovuo O Azurmendi Gil L Frantzén J van Gils M Admission Levels of Total Tau and β-Amyloid Isoforms 1-40 and 1-42 in Predicting the Outcome of Mild Traumatic Brain Injury. Oral Presentation at the 13th World Congress On Brain Injury (IBIA 2019). Toronto (2019).

[55] Hossain I Mohammadian M Takala RSK Tenovuo O Azurmendi Gil L Frantzén J van Gils M *Admission Levels of Total Tau and β*-Amyloid Isoforms 1-40 And 1-42 in Predicting the Outcome Of Mild Traumatic Brain Injury. Oral Presentation at the European Association of Neurosurgical Societies Annual Meeting (EANS 2019). Dublin (2019).

