# Supplementary material for: Admission Levels of Total Tau and β-Amyloid Isoforms 1–40 and 1–42 in Predicting the Outcome of Mild Traumatic Brain Injury
Source: Front Neurol. 2020 May 13;11:325. doi: 10.3389/fneur.2020.00325 (PMC7237639; doi:10.3389/fneur.2020.00325)
Supplement: Supplementary file 1 [file Table_1.DOCX]

eFigure 1. ROC curves for panels of biomarkers for predicting incomplete recovery (GOSE < 8). (Title)

A combination of the three biomarkers showed a higher sensitivity (92.5%) and specificity (27.8%) for predicting incomplete recovery compared to a single biomarker. (Caption)
