# Supplementary material for: Admission Levels of Total Tau and β-Amyloid Isoforms 1–40 and 1–42 in Predicting the Outcome of Mild Traumatic Brain Injury
Source: Front Neurol. 2020 May 13;11:325. doi: 10.3389/fneur.2020.00325 (PMC7237639; doi:10.3389/fneur.2020.00325)
Supplement: Supplementary file 2 [file Table_2.DOCX]

eFigure 2. Best multiparameter panel for outcome prediction. (Title)

The panel had a sensitivity of 90.8% and a specificity of 57.1% for predicting incomplete (GOSE < 8) recovery. (Caption)
