# Supplementary figures and images for: Admission Levels of Total Tau and β-Amyloid Isoforms 1–40 and 1–42 in Predicting the Outcome of Mild Traumatic Brain Injury
Source: Front Neurol. 2020 May 13;11:325. doi: 10.3389/fneur.2020.00325 (PMC7237639; doi:10.3389/fneur.2020.00325)

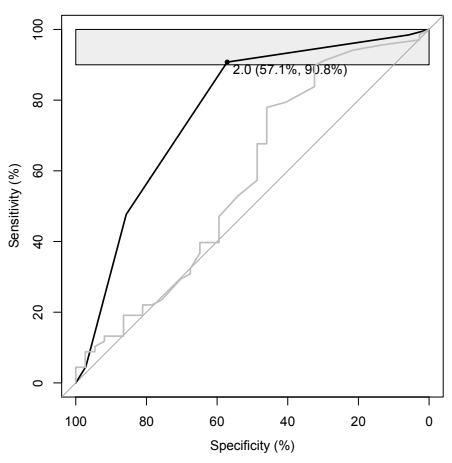

Supplement: Supplementary file 3 [file Image_1.TIF]

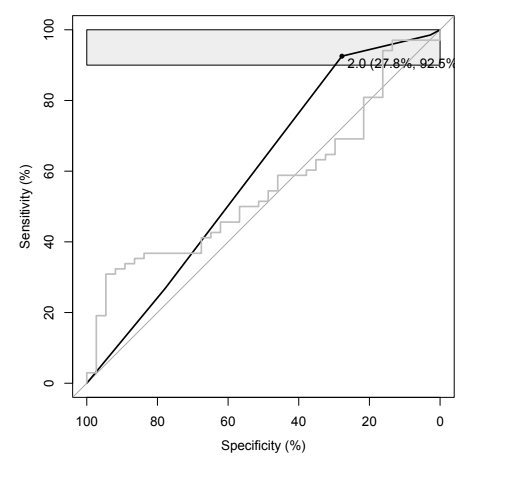

Supplement: Supplementary file 4 [file Image_2.TIF]
